# Development and characterization of an *in vitro* fluorescently tagged 3D bone-cartilage interface model

**DOI:** 10.3389/fendo.2024.1484912

**Published:** 2024-11-12

**Authors:** Mary Adams, Jessica Cottrell

**Affiliations:** ^1^ Department of Biological Sciences, Seton Hall University, South Orange, NJ, United States; ^2^ Immunology Translational Research, Translational Early Development, Bristol Myers Squibb, Summit, NJ, United States

**Keywords:** osteoblasts, osteoclasts, osteocytes, chondrocytes, cartilage, bone

## Abstract

Three-dimensional cultures are widely used to study bone and cartilage. These models often focus on the interaction between osteoblasts and osteoclasts or osteoblasts and chondrocytes. A culture of osteoblasts, osteoclasts and chondrocytes would represent the cells that interact in the joint and a model with these cells could be used to study many diseases that affect the joints. The goal of this study was to develop 3D bone-cartilage interface (3D-BCI) that included osteoblasts, osteocytes, osteoclasts, and cartilage. Fluorescently tagged cell lines were developed to assess the interactions as cells differentiate to form bone and cartilage. Mouse cell line, MC3T3, was labeled with a nuclear GFP tag and differentiated into osteoblasts and osteocytes in Matrigel. Raw264.7 cells transfected with a red cytoplasmic tag were added to the system and differentiated with the MC3T3 cells to form osteoclasts. A new method was developed to differentiate chondrocyte cell line ATDC5 in a cartilage spheroid, and the ATDC5 spheroid was added to the MC3T3 and Raw264.7 cell model. We used an Incucyte and functional analysis to assess the cells throughout the differentiation process. The 3D-BCI model was found to be positive for TRAP, ALP, Alizarin red and Alcian blue staining to confirm osteoblastogenesis, osteoclastogenesis, and cartilage formation. Gene expression confirmed differentiation of cells based on increased expression of osteoblast markers: *Alpl*, *Bglap*, *Col1A2*, and Runx*2*, cartilage markers: *Acan*, *Col2A1*, *Plod2*, and osteoclast markers: *Acp5*, *Rank* and *Ctsk*. Based on staining, protein expression and gene expression results, we conclude that we successfully developed a mouse model with a 3D bone-cartilage interface.

## 1 Introduction

Bone cell interactions are a fundamental aspect of bone physiology, critical for maintaining skeletal health and integrity. Within the network of bone tissue, various cell types collaborate to regulate bone remodeling, repair, and mineral homeostasis (1). Osteoblasts are responsible for bone formation and mineralization. Osteocytes are mature osteoblasts, embedded within the bone matrix, and play a central role in sensing mechanical signals and orchestrating the adaptive response of bone to external forces (2). Both osteoblasts and osteocytes communicate with osteoclasts, the bone-resorbing cells, to ensure a balance between bone resorption and new bone formation (3). These interactions are not only crucial for maintaining bone strength but are also integral to understanding bone-related diseases. Investigating the molecular and cellular mechanisms that govern bone cell interactions is essential for advancing knowledge of bone biology and developing innovative treatments for patients with bone disorders (4).

*In vivo* models are used for investigating bone and joint diseases, such as Rheumatoid Arthritis (RA). Inducible models were developed to mimic RA because animals used for *in vivo* testing do not naturally develop autoimmune disease. The most common model for testing RA is the collagen-induced arthritis model (CIA). However, the CIA model does not fully represent the complexity of the disease, has an unpredictable response, shows inconsistency between mouse strains and can only mimic short-term disease (4). Additionally, *in vivo* studies can be expensive, time consuming, and are often considered unethical. *In vivo* models can also give misleading safety data that does not correlate to human health and safety (5). As such, new legislation was passed in December 2022 by President Joe Biden that eliminates the need to test new medicines in animals to receive U.S. Food and Drug Administration (FDA) approval (6). Thus, there is a current need to develop new 3D models to confirm results from *in vitro* and *in vivo* experiments and reduce the use of animal models.

The development of 3D *in vitro* models is becoming increasingly prevalent as an effective way to reduce the overall number for animal studies (7, 8, 35–37). In the field of oncology, 3D models are being used to bridge the gap between 2D models and *in vivo* studies (9). 2D assays with cells growing on a flat surface do not accurately assess interactions in their natural, cellular environment and often show activity that is not recapitulated in animal models. Because 2D models do not directly correlate to activity, many potential therapeutics do not make it into clinical trials (10). It is also difficult to test multiple cell types together in a 2D model, and no such models have been developed to assess chondrocytes combined with bone cells. Some researchers are trying to fabricate a bone-to soft tissue interface while various studies document the need for a better model to study this cartilage-bone interface in a controlled but physiologically realistic setting (3, 13, 38–46). 3D models are more physiologically relevant than 2D cell culture. 3D models allow cells to maintain their physiological shape (2). 3D models include tissue explants, organoid and spheroid cultures, and microcarrier culture (8, 43, 47–49). Organoid cultures mimic specific tissues and involve multiple cell types grown together in an extracellular matrix such as hydrogel or Matrigel, which can allow for studying both cell-cell interaction and cell-matrix interactions (11). Organoid models can predict organ toxicity and can be used to measure drug activity (4). Patient derived organoids can increase the effectiveness of drug discovery, but cultures are time consuming, and often lack reproducibility (12). Hydrogel and Matrigel biomaterials are currently being used as bioinks to allow for 3D printing of bone (11, 50). A successful 3D osteochondral tissue model was generated by Damerau, et al., that combined differentiated chondrocytes and osteoblasts to assess an inflammatory response on bone and cartilage (13). This model mimicked cytokine-induced cellular and matrix-related changes of cartilage degradation. Unfortunately, since the model did not include both osteoblasts and osteoclasts it could not assess bone remodeling process. Additionally, this model omitted specific RA-related leukocyte population and/or endothelial cells so cellular behavior, and intracellular interactions could not be addressed. Overall, this model was a good starting point but did not have many components, which are necessary for bone growth and remodeling (3, 48).

Many scientists are also developing organ-on-a-chip platforms (14). Organ-on-a-chip platforms are microfluid platforms that allow for controlling oxygen and nutrients that require very few cells and can assess cell-cell interactions (15). Cartilage-on-a-chip models have been developed that embed chondrocytes into hydrogel; these models can apply pressure and induce inflammatory responses to mimic diseases such as RA and Osteoarthritis (OA). Bone-on-a-chip models have also been developed that include osteoblasts and osteoclasts and endothelial cells. There is a possibility of combining cartilage and bone chips to develop a joint-on-a chip, though due to the extreme complexity of the cartilage and bone interactions, this has not yet been developed (16, 51, 52). These organ-on-a-chip models keep each cell type spatially separated and cannot visualize the interplay of the cells through bone formation and homeostasis. The dysregulation of osteoclast and osteoblast homeostasis in RA drives the disease, as does the destruction of the cartilage, so assessing the interaction between the osteoblasts, osteoclasts and chondrocytes in the joint is critical (14, 32–34).

None of the current *in vitro* models that have been developed assess the interplay between osteoblasts, osteoclasts and chondrocytes. These interactions are essential for studying diseases that involve the joints (12). To address this gap, we have developed a mouse 3D bone-cartilage interface (3D-BCI) model that includes fluorescently tagged osteoblast, osteoclasts, and chondrocyte cell lines so that we can visualize the interaction of the cells as they differentiate into bone and cartilage and measure protein and gene expression changes in a model containing osteoblasts, osteoclasts and chondrocytes. In developing this 3D-BCI model we have also established a novel way to differentiate ATDC5 cells in a 3D cartilage spheroid. This method is cost effective and less labor intensive than differentiating primary chondrocytes into cartilage. The 3D-BCI model and 3D ATDC5 spheroids could potentially be used to study diseases of the joint such as RA and osteoarthritis

## 2 Materials and methods

### 2.1 Cell culture

Raw264.7 cells were obtained from ATCC (TIB-71, Manassas, VA) and cultured DMEM (11965-084, Gibco, Waltham, MA) containing 10% FBS (16000-044 Gibco, Waltham, MA). MC3T3-E1 Subclone 4 cells were obtained from ATCC (CRL-2593, Manassas, VA) and cultured MEM Alpha (12571-063 Gibco, Waltham, MA) containing 10% FBS. ATDC5 cells were obtained from Millipore Sigma (99072806, Burlington, MA) and cultured in DMEM/F-12 (11320033, Gibco, Waltham, MA) containing 5% FBS and L-glutamine (A2916801, Gibco, Waltham, MA). All cells were maintained at 37°C with 5% CO2 and 95% Relative Humidity.

### 2.2 Transfections

Nuclight Green Lentivirus (4624 Sartorius, Bohemia, NY) and Cytolight Red Lentivirus (4481, Sartorius, Bohemia, NY), containing EF1 alpha promoter region and puromycin selection, were used to transfect cells. Cells were cultured for 1-2 weeks and transfections were performed at passage 2-3. A total of 1.5x10^5^ cells were plated in 150 µl of media into a 48 well plate. Cells were incubated for 24 hours to allow attachment. A 4x solution of Lentivirus (MOI 3, 6 and 9 Titer Units/cell) in media was made with 8 µg/mL Polybrene (TR-1003, Millipore Sigma, Burlington, MA). A total of 50 µl of media containing virus was added to cells and cells were incubated for 24 hours. The virus was removed, and cells were incubated for recovery for 48 hours before adding 3 µg/mL puromycin (P9620, Millipore Sigma, Burlington, MA). Confirmation of transfection was performed through Incucyte Image analysis (4647, Sartorius, Bohemia, NY). Note: The Raw264.7 cell transfection after troubleshooting, was successful without polybrene and required a 10-minute centrifugation step after lentivirus was added to the cells. Cells were grown until the cells were confluent, cells were then split and grown in T25 flasks. Cells were scaled up over a one-month period and then stocks of transfected cells were frozen down and stored in liquid nitrogen dewars.

### 2.3 Staining techniques

Alcian Blue 8GX (A3157, Millipore Sigma, Burlington, MA) was used to detect proteoglycan accumulation. An Alcian blue solution was prepared with 75% EtOH and 0.1M HCl (4:1). Cells were fixed with methanol for 10 minutes and washed with PBS. Alcian blue was added and incubated overnight at 37°C. Wells were washed 3 times with DI water. Alizarin Red (A5533, Millipore Sigma, Burlington, MA) was used to measure mineralization. Cells were fixed with methanol for 10 minutes and washed with PBS. A 4% Alizarin red solution was added, and plates were incubated at room temperature for 30 minutes. The Alizarin Red was removed, and cells were washed 3 times with DI water. Tartrate-resistant acid phosphatase (TRAP) was used as an osteoclast marker. Cells were washed with PBS and fixed with 4% paraformaldehyde and TRAP stained (MK301, Takara, San Jose, CA) according to manufacturer’s protocol. All Images were captured with the EVOS microscope (M7000, ThermoFisher, Waltham, MA).

### 2.4 Osteoblast differentiation

For the 2D differentiation, MC3T3 cells were plated on 12-well plates at 2.5x10^5^ cells/well in 500 µL media. Cells were incubated until confluent. Osteoblast differentiation media (OBM) was prepared with MEM Alpha, 10% FBS, 100 µM L-Ascorbic acid-2-Phosphate (49752, Millipore Sigma, Burlington, MA), 2 mM β-glycerophosphate (G9422, Millipore Sigma, Burlington, MA), 100 nM Dexamethasone (D4902, Millipore Sigma, Burlington, MA), and 100 ng/mL BMP-2 (355-BM, RD Systems, Minneapolis, MN). Media was aspirated from cells and replaced with 500 µL differentiation media. Half of the differentiation media was replaced every 3-4 days. Cells were incubated for 28 days. On Days 7, 14, and 21 cells were washed with 1 mL PBS, fixed with 500 µL methanol and stored at -20°C. On Day 28 the final plate was washed, and all plates were stained with Alizarin Red.

### 2.5 Chondrocyte differentiation

For 2D differentiation, ATDC5 cells were plated on 12-well plates at 2.5x10^5^ cells/well in 500 µL media. Cells were incubated until confluent (2-3 days). Chondrocyte differentiation media was prepared with DMEM/F-12, 5% FBS, L-glutamine and 1% ITS (insulin, transferrin, and sodium selenite, I3146 Millipore Sigma, Burlington, MA). Media was aspirated from cells and replaced with 500 µL differentiation media. Half of the differentiation media was replaced every 3-4 days. Cells were differentiated over 28 days. On Days 7, 14, and 21 cells were washed with 1 mL PBS, fixed with 500 µL methanol, and stored at -20°C. On Day 28 all plates were stained with Alizarin Red or Alcian Blue 8GX. For 3D differentiation, ATDC5 cells were plates on 96-well Ultra Low Attachment (ULA, 7007, Corning, Corning, NY) plates at 4x10^4^ cells/well in 100 µL media. Cells were centrifuged at 200 RCF for 10 minutes to form spheroids. Differentiation media, 100 µL, 2X concentration was added to the pelleted cells and cells were incubated for 21 days with sphere size measured every 24 hours with the Incucyte. Half of the differentiation media was exchanged every 2-3 days. Spheroid differentiation was measured with Alizarin Red and Alcian Blue.

### 2.6 Osteoclast differentiation

Raw264.7 cells were plated on 96-well plates at 5x10^3^ cells/well in 100 µL of media. Cells were incubated overnight to allow attachment. Osteoclast differentiation media (OCM) was prepared with DMEM, 10% FBS, and 100 ng/mL RANKL (462-TEC-010, RD Systems, Minneapolis, MN). Media was aspirated from cells and replaced with 200 µL differentiation media. Half of the differentiation media was replaced every 2-3 days. On day 7 cells were TRAP stained.

### 2.7 3D joint model

For 3D differentiation, MC3T3 cells were counted and mixed with growth factor reduced Matrigel (354230 Corning, Corning, NY) at a concentration of 6.0x10^4^ cells/17 µl. Droplets of cells were carefully applied to 96-well ULA plates. Plates were incubated at 37°C for 5 minutes to allow polymerization of Matrigel. OBM (200 µL) was added to the wells and cells were incubated for 21 days to allow for mineralization. Half of the OBM was changed every 3-4 days. Cells were imaged on the Incucyte. On Day 21, Raw264.7 cells were added to the MC3T3 cells at 6.0 x10^4^ cells/well. RANKL (100 ng/mL) was added to the OBM. Half of the OBM was changed every 3-4 days. On Day 28 ATDC5 Spheroids (at Day 21 of differentiation) were transferred to the 3D bone using wide bore tips (2069G, ThermoFisher, Waltham, MA). The 3D joint was incubated in 200 µL OBM + RANKL for an additional 7 days, replacing half the media every 2-3 days. Cell supernatant was collected, and protein expression was assessed using a mouse Luminex assay (Millipore Sigma, Burlington, MA). Cell differentiation was measured by ALP, Alizarin Red, Alcian Blue and TRAP staining. Excel was used to perform the protein expression data analysis and p Values were calculated using GraphPad Prism unpaired t tests.

### 2.8 RT-PCR

Separate samples of undifferentiated MC3T3, ATDC5 and Raw264.7 cells, along with 3D-BCI samples containing all three cell types were lysed in buffer RLT (79216, Qiagen, Germantown, MD) and RNA was isolated using the RNeasy Mini Kit (74104, Qiagen, Germantown, MD) with the addition of the RNase-free DNase kit (79254, Qiagen). The nanodrop was used to measure RNA concentration and RNA was converted to cDNA using SuperScript™ IV VILO™ Master Mix (11756050, Applied Biosystems, Carlsbad, CA). All primers in **Table 1** were purchased from IDT (Coralville, IA). RT-PCR was performed on the Quantstudio Pro using PowerUp™ SYBR™ Green Master Mix (A25742, Applied Biosystems, Carlsbad, CA). Osteoblast gene expression was assessed with *Alpl*, *Bglap*, *Col1a1*, *Mmp2*, *Runx2* and *Sparc*. *B2m* was used as the housekeeping gene. Relative quantification was calculated as 2-ΔΔCT with the first ΔCT to the *B2m*M housekeeping gene and the second ΔCT to the undifferentiated MC3T3 cells. Osteoclast gene expression was assessed with *Acp5*, *Ctsk*, *Dcstamp*, *Ocstamp* and *Rank*. Relative quantification was calculated as 2-ΔΔCT with the first ΔCT to the *B2m* housekeeping gene and the second ΔCT to the undifferentiated Raw264.7 cells. Cartilage gene expression was assessed with *Acan*, *Col2a1*, *Crtap*, *Plopd2* and *Sox9*. Relative quantification was calculated as 2-ΔΔCT with the first ΔCT to the *B2m* housekeeping gene and the second ΔCT to the undifferentiated ATDC5 cells. Excel was used to perform gene expression data analysis and p Values were calculated using GraphPad Prism one-way ANOVA.

**Table 1:** Quantitative Real-time PCR Primer Sets.

Gene Symbol	IDT Assay ID	Ref Seq #	Full Gene Name
*B2m*	Mm.PT.39a.22214835	NM_009735	Beta-2 Microglobulin
*Crtap*	Mm.PT.58.13597418	NM_019922	Cartilage-associated protein
*Runx2*	Mm.PT.58.41866893	NR_073425	Runt-related transcription factor 2
*Calcr*	Mm.PT.58.13013231	NM_001042725	Calcitonin Receptor
*Col1a2*	Mm.PT.58.5206680	NM_007743	Collagen Type I Alpha 2 Chain
*Bmp7*	Mm.PT.58.42850153	NM_007557	Bone Morphogenetic Protein 7
*Acan*	Mm.PT.58.10174685	NM_007424	Aggrecan
*Alpl*	Mm.PT.58.8794492	NM_007431	Alkaline Phosphatase, Biomineralization Associated
*Mmp2*	Mm.PT.58.9606100	NM_008610	Matrix Metallopeptidase 2
*Tnfrsf11a*	Mm.PT.58.8027089	NM_009399	receptor activator of NF-kB (RANK)
*Ctsk*	Mm.PT.58.10366461	NM_007802	Cathepsin K
*Dcstamp*	Mm.PT.56a.32438320	NM_029422	Dendrocyte Expressed Seven Transmembrane Protein
*Plod2*	Mm.PT.58.14020153	NM_001142916	Procollagen-Lysine,2-Oxoglutarate 5-Dioxygenase 2
*Acp5*	Mm.PT.58.5755766	NM_001102404	Acid Phosphatase 5 (TRAP)
*Spp1*	Mm.PT.58.43709208	NM_001204201	Secreted Phosphoprotein 1
*Sparc*	Mm.PT.58.17039746	NM_009242	Secreted Protein Acidic and Cysteine Rich
*Col2a1*	Mm.PT.58.10123677	NM_031163	Collagen Type II Alpha 1 Chain
*Bmp2*	Mm.PT.58.10419414	NM_007553	Bone Morphogenetic Protein 2
*Ocstamp*	Mm.PT.56a.43459025	NM_029021	Osteoclast Stimulatory Transmembrane Protein
*Sox9*	Mm.PT.58.42739087	NM_011448	SRY-Box Transcription Factor 9
*Bglap*	Mm.PT.58.9119501.g	NM_001037939	Bone Gamma-Carboxyglutamate Protein

## 3 Results

### 3.1 Raw264.7 cells, MC3T3 cells and ATDC5 cell lines were successfully transfected with fluorescent tags

**Figure 1:**
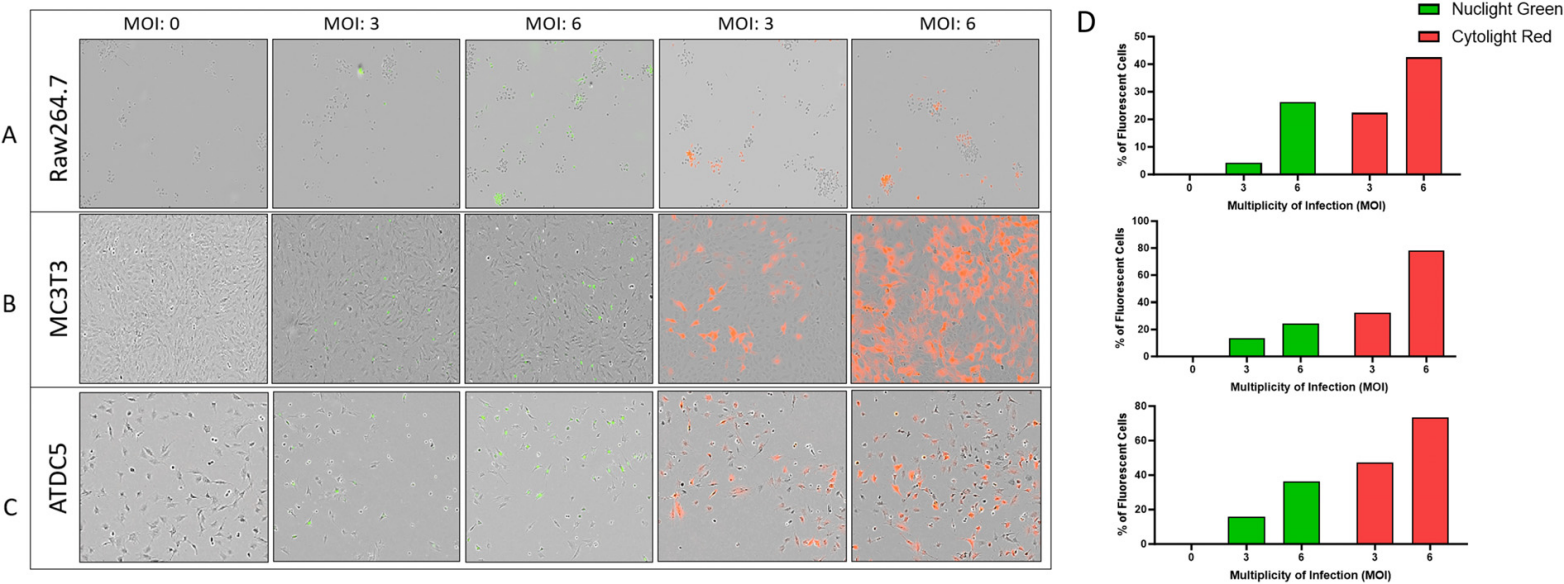
Transfection efficiency of Raw264.7, MC3T3, and ATDC5 transfections with Nuclight Green or Cytolight Red. Images of the cells 5 days post-transfection were taken with the Incucyte at 20x magnification. **(A–C)** represent images of Raw264.7, MC3T3 and ATDC5 respectively, based on Multiplicity of Infection (MOI). **(D)** The transfection efficiency was calculated for each MOI for each cell line. Transfection was performed only once per cell line.

To assess the interaction of the cells in the 3D model, Raw264.7, MC3T3, and ATDC5 cell lines were transfected with either nuclear green or cytoplasmic red fluorescent tags and were visualized with the Incucyte live cell imager. The higher titers of virus were more successful in transfection efficiency than the lower Multiplicity of Infections (MOI) tested. An MOI of 1 was also tested for each lentivirus but there was little to no transfection efficiency (data not shown). In Raw264.7, MC3T3 and ATDC5 cells, the transfection efficiency for Nuclight Green (MOI 6) was 26%, 24%, and 36% respectively compared to 42%, 78%, and 73% for Cytolight Red (MOI 6, **Figures 1A–D**). The ATDC5 cells transfected with Nuclight Green did not survive the transfection and the cell line could not be further propagated. In **Figure 1C** it is clear the cells were not healthy, and their morphology was more rounded than the ATCD5 parental cell line.

### 3.2 Lentivirus transfections did not interfere with cell proliferation or differentiation

**Figure 2:**
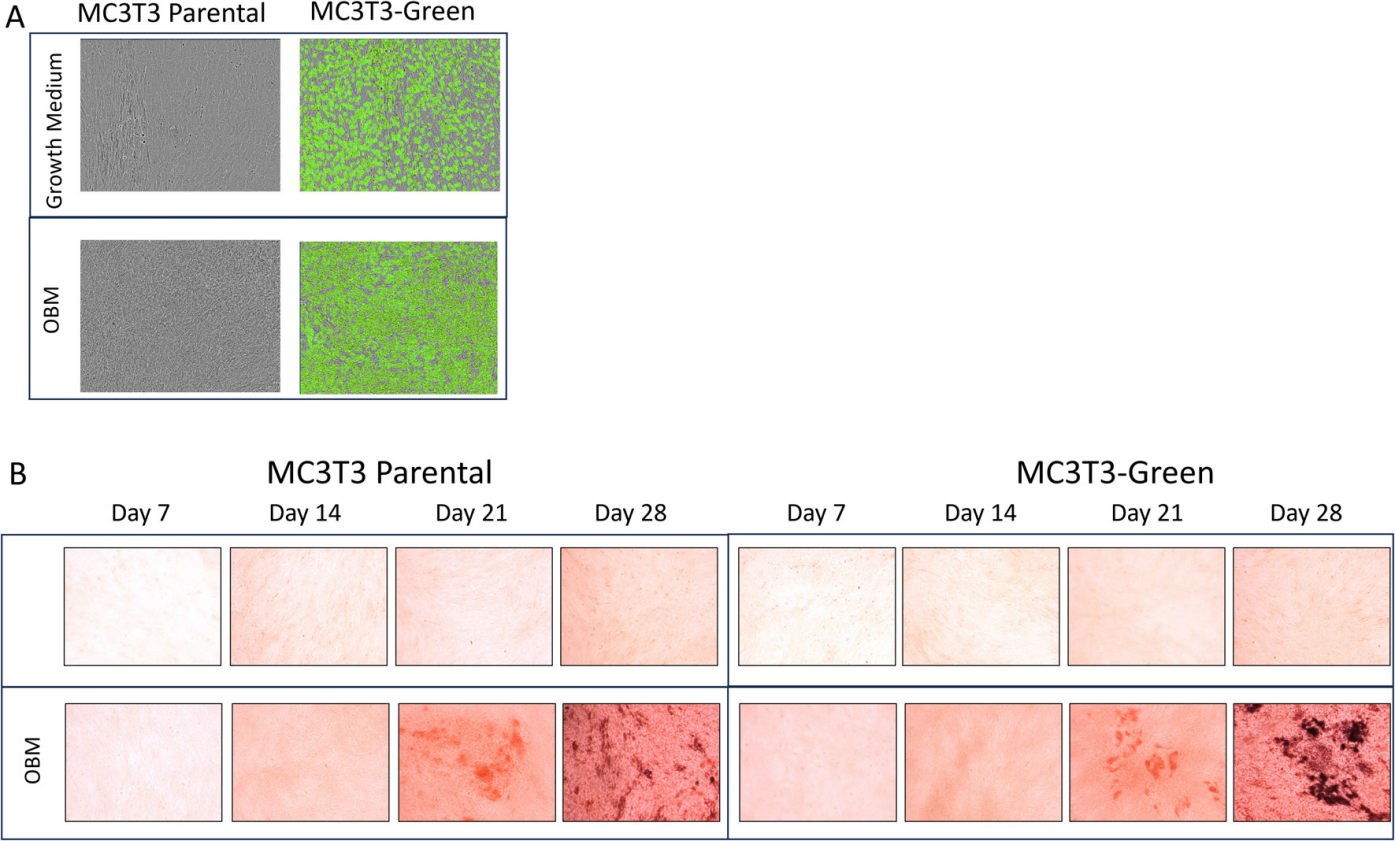
2D Osteoblast differentiation of transfected MC3T3 cells. **(A)** MC3T3 parental and MC3T3-Green cell lines were differentiated with osteoblast differentiation medium (OBM) for 14 days and then images were taken on the Incucyte to capture the fluorescence. **(B)** MC3T3 parental and MC3T3-Green cell lines were differentiated with OBM for 28 days and then cells were stained with Alizarin Red. All colorimetric images were captured on the EVOS microscope. Differentiation assays were repeated three times.

After transfecting Raw264.7 cells, MC3T3 cells and ATDC5 cell lines, 2D differentiation assays were performed to assess differentiation ability of the transfected versus parental cell lines to confirm they will differentiate in the 3D-BCI model. **Figure 2A** shows MC3T3 Red cells treated with OBM for 14 days. On day 14, there was similar calcification visible in the MC3T3-Red cells when compared to parental cell lines via Alizarin staining. Over the course of 28 days, the MC3T3-Green cell line increased mineralization when compared to the MC3T3-Parental. Both the MC3T3-Green and MC3T3-Red transfected cell line behaved similarly to the parental line in both growth rates and mineralization (**Figure 2B**).

**Figure 3:**
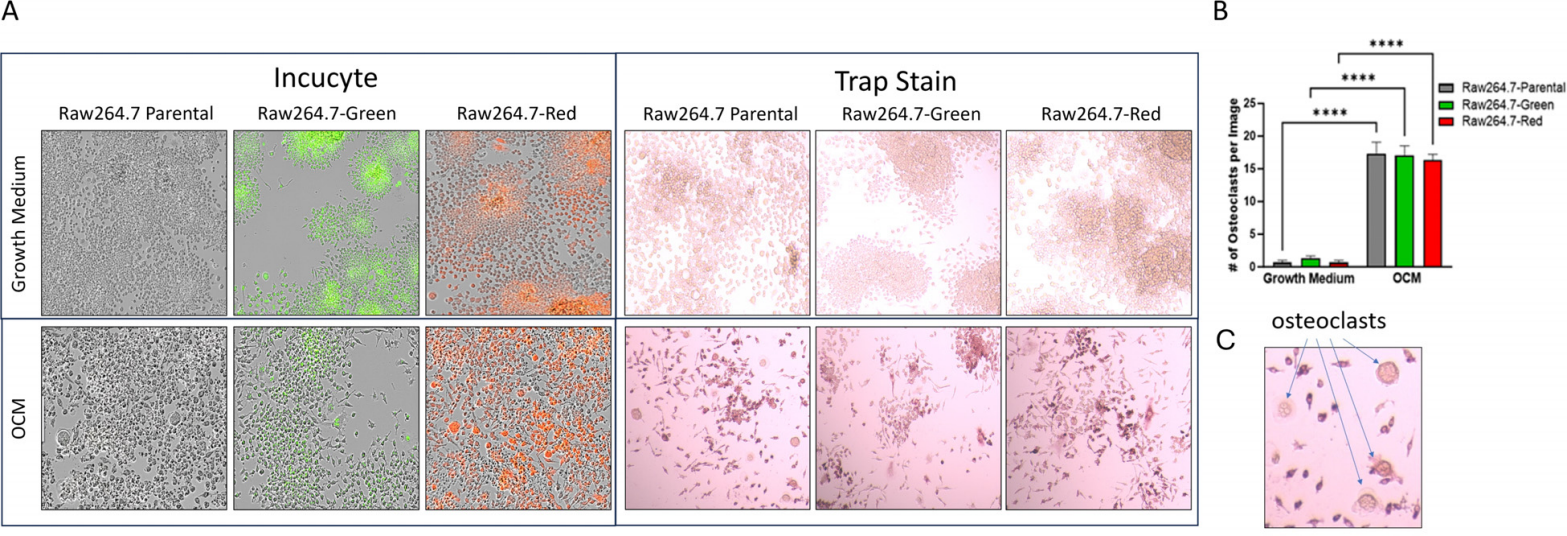
2D Osteoclastogenesis of Raw264.7 cells. **(A)** Raw264.7 parental, Raw264.7-Green and Raw264.7-Red cell lines were differentiated with osteoclast differentiation medium (OCM) for 7 days. Cells were imaged on the Incucyte (Left) and then TRAP stained (Right). Colorimetric images were captured on the EVOS microscope. **(B)** After the 7-day differentiation, TRAP positive osteoclasts (containing three or more nuclei) were quantified. **(C)** Enlarged image of osteoclasts. The graph represents n=3. Statistical analysis was performed using GraphPad Prism one-way ANOVA. ****p < 0.0001. No statistical difference was found between the differentiated Raw 264.7 cell groups, p=0.89.

Raw264.7 parental, Raw264.7-Green and Raw264.7-Red cell lines were successfully differentiated into osteoclasts as evidence by TRAP staining (**Figure 3A**). When evaluating these cell lines, no significant differences were found in TRAP positive cells after 7 days p=0.89 (**Figure 3**).

**Figure 4:**
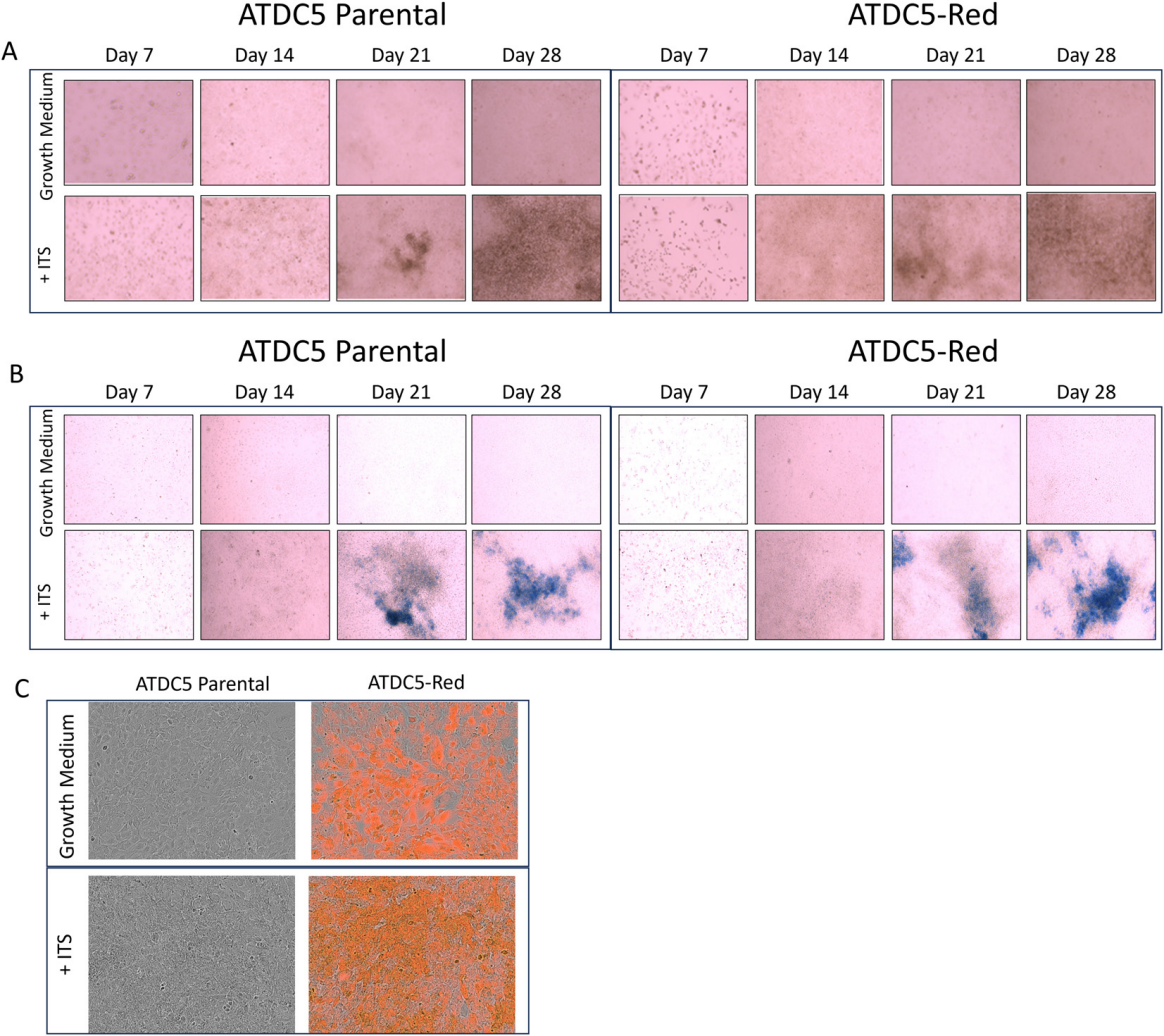
2D Chondrocyte Differentiation of transfected ATCD5 cells. ATDC5 parental and ATDC5-Red cell lines were differentiated with Insulin, Transferrin and Selenium (ITS) for 28 days. Cells were stained at Days 7, 14, 21 and 28 for Alizarin Red **(A)** and Alcian Blue **(B)**. **(C)** Cells were imaged on the Incucyte on Day 28 to visualize fluorescence and mineralization patterns. Differentiation assays were repeated three times.

ATDC5-parental and ATDC5-Red cells were differentiated for 28 days. Alizarin Red (**Figure 4A**) and Alcian Blue staining (**Figure 4B**) demonstrated that both cells were able to deposit calcium and proteoglycans similarly. **Figure 4C** shows similar mineralization patterns for ATDC5-parental and ATDC5-Red on Day 28 post ITS treatment.

### 3.3 ATDC5 cell differentiation into 3D cartilage spheroids

**Figure 5:**
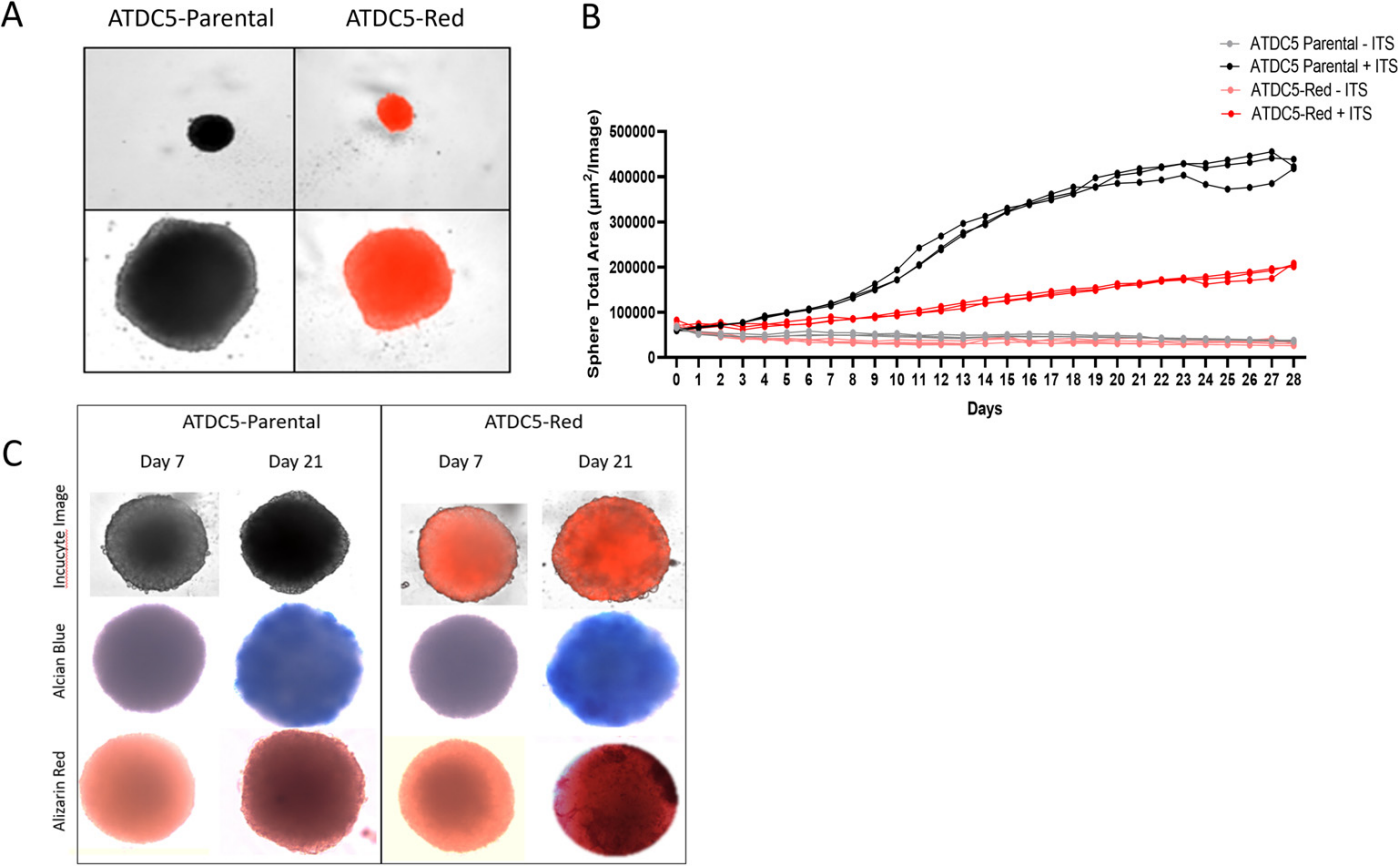
3D ATDC5 Spheroid Formation. **(A)** ATDC5-parental and -red cells were plated in ultra-low attachment (ULA) plates, centrifuged to form spheroids and differentiated for 21 days. **(B)** During the differentiation, the Incucyte measured spheroid size every 24 hours for 28 days. **(C)** ATDC5-parental and -red spheres were differentiated for 7 or 21 days and fluorescent images were taken on the Incucyte. Spheres were then stained for Alcian Blue and Alizarin Red. Colorimetric images were captured on the EVOS microscope. Spheroid differentiation assays were repeated three times.

**Figure 6:**
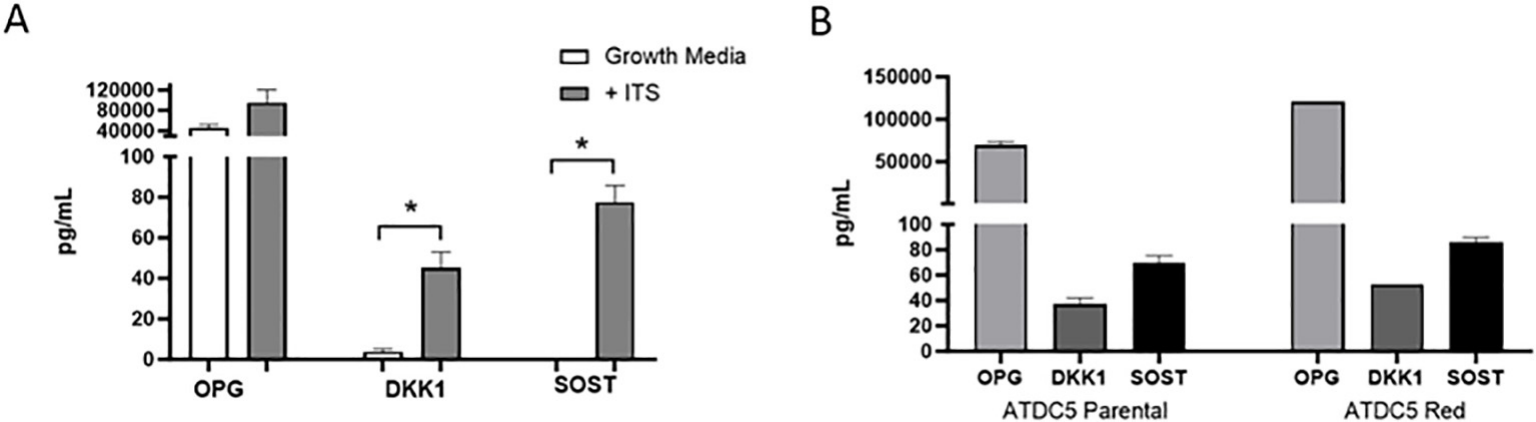
3D Chondrocyte Spheroid Protein Expression. ATDC5-parental and -red cells were plated in ULA plates, centrifuged to form spheroids and differentiated for 21 days. Supernatant was collected and OPG, DKK1 and SOST protein levels were measured by Luminex assay. **(A)** Comparison of protein levels released by 3D chondrocytes grown in growth media without ITS versus differentiation media containing ITS. **(B)** Protein level comparison of ATDC5 parental versus ATDC5-Red transfected cells after the 21-day differentiation with ITS. Statistical analysis was performed using Graphpad Prism unpaired t-tests. *p < 0.05, n=3.

ATDC5 parental and ATDC5-Red cell lines were grown in ULA plates with ITS to develop a 3D cartilage tissue suitable for integration into the 3D bone model containing differentiated osteoblasts and osteoclasts (**Figure 5A**). Both ATDC5 parental and ATDC5-Red spheroids consistently grew over the course of 21 days (p=0.0057, **Figure 5B**). Both spheroids stained positively for calcium and GAGs (**Figure 5C**). Based on staining results, the ATDC5 cells were fully differentiated into cartilage by Day 21. On Day 21 supernatant was removed from the spheres and protein levels of Osteoprotegerin (OPG), Dickkopf WNT signaling pathway inhibitor 1 (DKK1) and Sclerostin (SOST) were measured. There was a 12- and 78-fold increase in DKK1 and SOST protein expression, respectively, in differentiated spheroids compared to undifferentiated spheroids grown in growth media. There was no significant difference in protein levels released by parental versus transfected ATDC5 spheroids (p>0.99, **Figure 6**).

**Figure 7:**
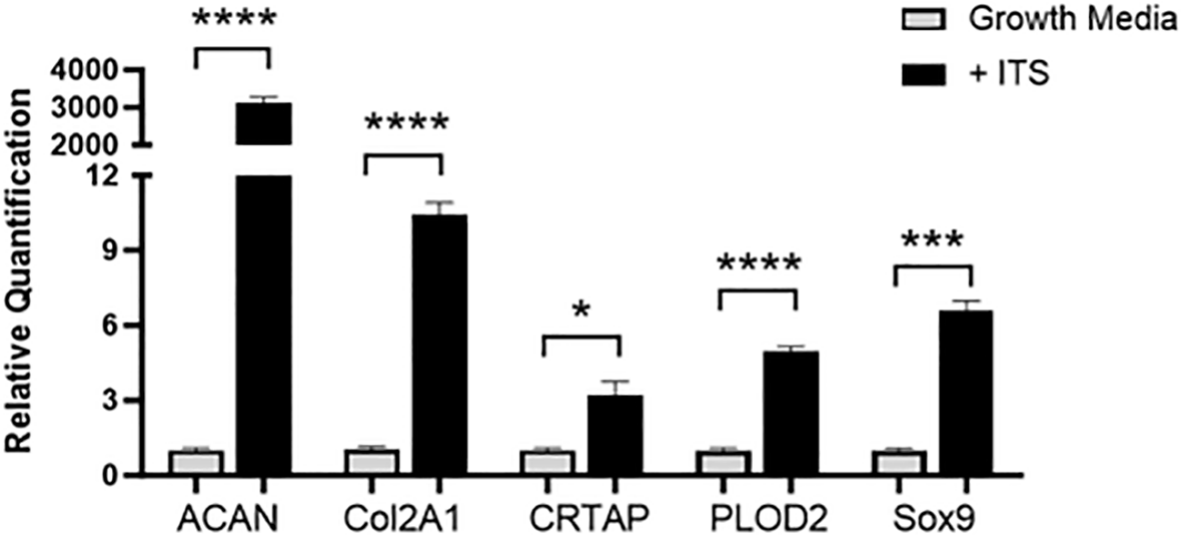
3D Chondrocyte Gene Expression. ATDC5-parental and -red cells were plated in ULA plates, centrifuged to form spheroids and differentiated for 21 days. On Day 21 cells were lysed, RNA was extracted and converted to cDNA and RT-PCR was performed to assess gene expression changes in cartilage markers ACAN, Col2A1, CRTAP, PLOD2 and Sox9. Relative quantification (RQ) was measured by normalizing to B2M housekeeping gene and then normalizing each gene to the undifferentiated ATDC5 samples. Statistical analysis was performed using Graphpad Prism unpaired t-tests. *p < 0.05; ***p < 0.001; ****p < 0.0001, n=3.

RT-PCR was run with cartilage gene markers to confirm the ATDC5 chondrocytes differentiated into cartilage. Cartilage markers *Acan*, *Col2a1*, *Crtap*, *Plod2* and *Sox9* were measured. **Figure 7** shows that all cartilage markers significantly increased in the 21-day spheres grown with ITS as compared to the undifferentiated ATDC5 cells.

**Figure 8:**
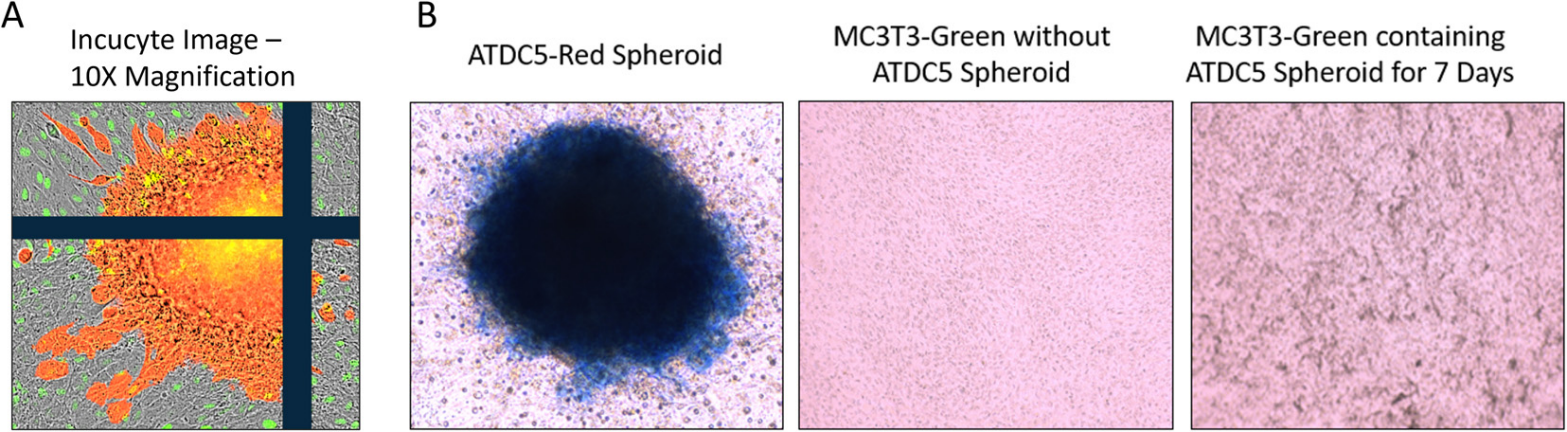
3D ATDC5 spheroid successfully attached to 2D differentiated osteoblasts. A ATDC5-Red spheroids were differentiated with ITS for 7 days and then one spheroid was transferred to a plate containing 14-day 2D differentiated MC3T3-Green cells. **(A)** Incucyte visualization of the ATDC5-Red spheroid attached 7 days after being transferred to the 2D differentiated MC3T3-Green cells. **(B)** The well containing the osteoblasts and the spheroid positively stained for Alcian blue, indicating the spheroid contained glycosaminoglycans (GAGs). The MC3T3-Green cells in the same well also had positive Alcian blue staining while a separate well of differentiated MC3T3-Green cells without the ATDC5-Red spheroid did not stain positive for GAGs.

Once ATDC5 spheroids were confirmed to have cartilage properties, they were plated with osteoblasts to assess attachment and integration. A 7-day old ATDC5-Red spheres were placed in a 2D MC3T3-Green differentiation plate and grown in OBM for an additional 7 days. **Figure 8** shows that the sphere successfully attached to the osteoblast surface and started to spread as observed by the movement of fluorescently tagged ATDC5 cells. Alcian Blue staining of the ATDC5-Red spheroid/MC3T3-Green plate was positive for glycosaminoglycans (GAGs). The MC3T3 Green Containing ATDC5 Spheroid on Day 7 image of the farthest location in the well from the spheroid also tested positive for GAGs, indicating that the ATDC5 spheroid was affecting the MC3T3 cells in the entire well.

### 3.4 Three-dimensional differentiation of bone and cartilage

To create a 3D bone-cartilage interface (3D-BCI), ATDC5 spheroids were combined with 3D-bone organoids. MC3T3 parental and MC3T3-Red cell lines were embedded in Matrigel and grown in OBM for 21 days. Once clear mineralization was observed, Raw264.7 parental or Raw264.7-Red cells were added to the differentiated osteoblasts. RANKL was added to the OBM and osteoclastogenesis was induced for 7 days. On Day 28 ATDC5 parental or ATDC5-Red spheroids were added to the 3D bone and the cells were grown in differentiation media containing OBM + RANKL for an additional 7 days, forming the 3D-BCI.

**Figure 9:**
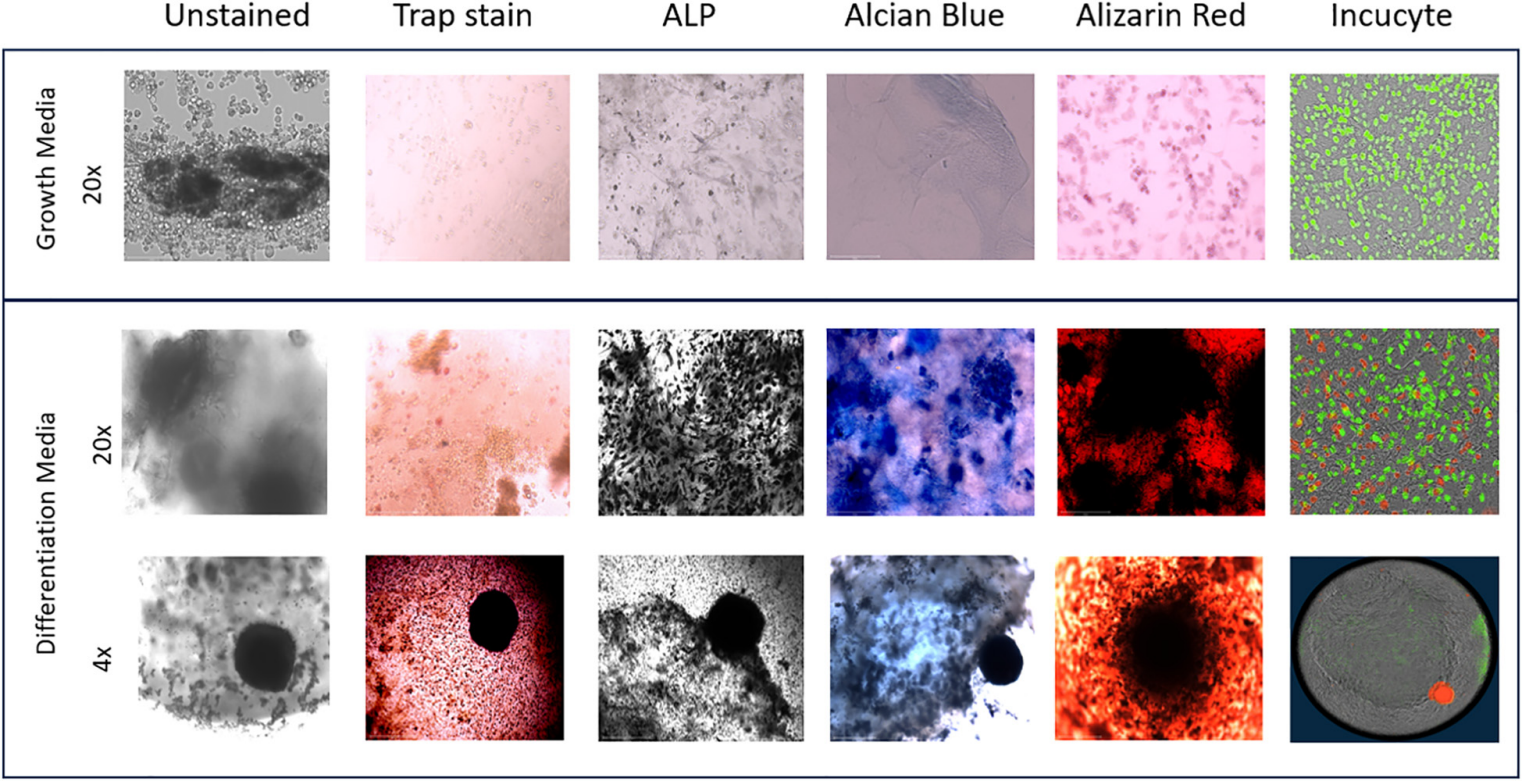
3D Bone Cartilage Interface Image. MC3T3 cells were plated in Matrigel and differentiated with OBM for 21 days. On Day 21 Raw264.7 cells were added to the 3D culture and differentiated for an additional 7 Days. On Day 28 ATDC5 spheroids were added to the 3D culture and differentiated for an additional 7 Days. Fluorescent images were taken on the Incucyte. Cells were then removed from the Incucyte and fixed and stained with TRAP, ALP, Alcian blue or Alizarin Red. Unstained and stained images were taken with the EVOS microscope. All 20x images were taken of areas without the spheroid while the 4x images were taken of the 3D joint containing the spheroid. n=3.

On day 36, images of the 3D-BCI found that Raw264.7 cells were directly interacting with the MC3T3 cells (**Figure 9**). Unstained images revealed osteoid formation and ATDC5-red spheroid attachment (4x magnification). The 3D bone and ATDC5 spheroid both stained positive TRAP, ALP, Alizarin Red, and Alcian Blue.

**Figure 10:**
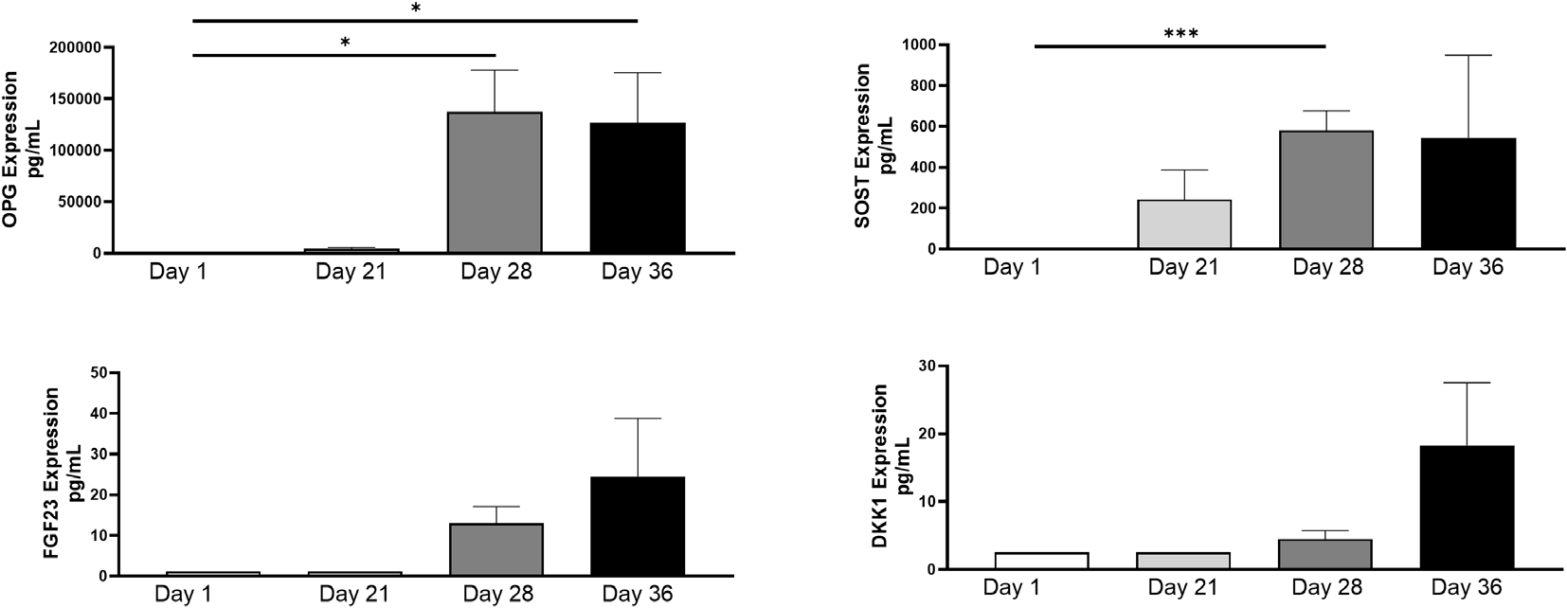
3D Bone Cartilage Interface Protein Expression. MC3T3 cells were plated in Matrigel and differentiated with OBM for 21 days. On Day 21 Raw264.7 cells were added to the 3D culture and differentiated for an additional 7 Days. On Day 28 ATDC5 spheroids were added to the 3D culture and differentiated for an additional 7 Days. Supernatant was collected on Day 1, Day 21, Day 28 and Day 36. Protein levels were measured via Luminex assay. Statistical analysis was performed using Graphpad Prism unpaired t-tests. *p < 0.05, ***p < 0.001, n=3.

Protein levels were isolated from collected spent supernatant of the 3D-BCI model on days 1, 21, 28 and 36. OPG and SOST were 25015% and 29048% significantly increased in the 3D-BCI model by Day 28 when compared to Day 1 (OPG p=0.0486 and SOST p=0.0003, **Figure 10**). FGF23 and DKK1 showed increased expression by day 36 but was not found to be statistically significant.

**Figure 11:**
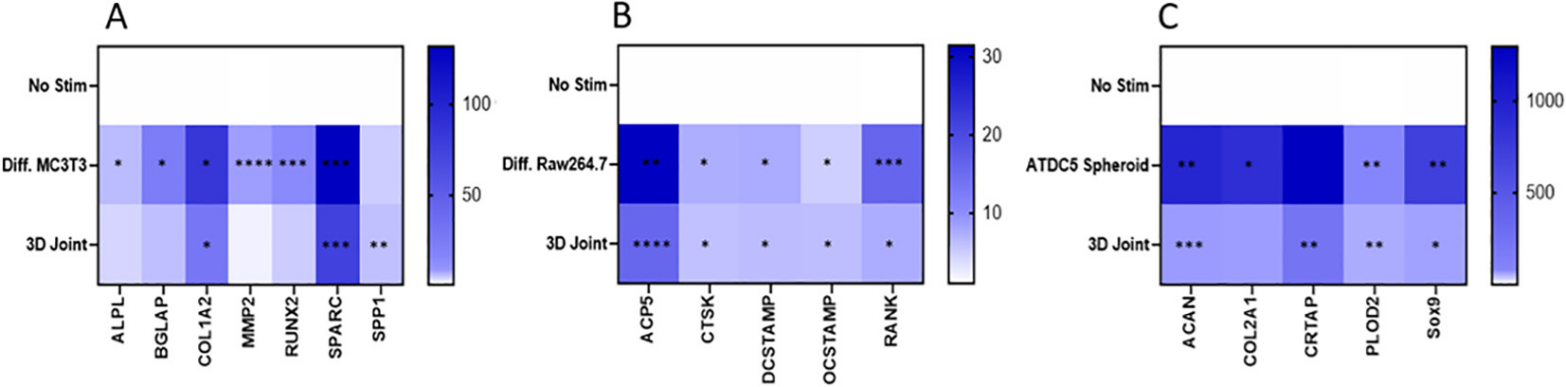
3D Bone-Cartilage interface gene expression. On Day 36 cells were lysed, RNA was extracted, converted to cDNA, and RT-PCR was performed to assess gene expression changes in osteoblast, osteoclast, and cartilage markers. Relative quantification (RQ) was measured by normalizing to B2M housekeeping gene and then normalizing each gene to the No Stim, undifferentiated cells. Statistical analysis was performed using Graphpad Prism one-way ANOVA. *p < 0.05; **p < 0.01; ***p < 0.001; ****p < 0.0001, n=3. No Stim, No Stimulation; Diff., Differentiated.

Genes expression was measured in undifferentiated MC3T3 cells and 3D differentiated MC3T3 cells, undifferentiated and differentiated Raw264.7 cells, undifferentiated and 3D differentiated ATDC5 cell, and the combined cells of the 3D BCI model on Day 36 (**Figure 11**). Gene expression for each cell type was normalized separately using housekeeping genes and the no stimulation control cells for the specific cell type. RQ values for stimulated samples and BCI model samples were then calculated based on the RQ value of unstimulated sample for the specific cell type being equal to 1. The list of primers tested is located in **Table 1** and contains osteoblast, osteoclast, and cartilage markers. The 3D differentiated MC3T3 cells showed > 391% significant increased expression for *Alpl*, *Bglap*, *Col1a1*, *Mmp2*, *Runx2* and *Sparc* when compared to undifferentiated MC3T3 cells (p=0.0004, **Figure 11A**). The 3D BCI model had >488% significantly increased expression of *Col1a1*, *Sparc* and *Spp1*(p=0.0018). The other osteoblast markers were increased in the 3D BCI model, but results were not significant. All osteoclast markers were >485% significantly increased in both the differentiated Raw264.7 cells and the 3D joint model when compared to the undifferentiated Raw264.7 cells (**Figure 11B**, p=0.0348). *Acan*, *Col2a1*, *Plod2* and *Sox9* were >6439% significantly increased in the ATDC5 spheroid when compared to undifferentiated ATDC5 cells (p=0.0099, **Figure 11C**). *Acan*, *Crtap*, *Plod2* and *Sox9* were >653% significantly increased in the 3D BCI model when compared to undifferentiated ATDC5 cells (p=0.0093). Differentiation markers all had lower expressions in the 3D BCI samples, which contain a combination of MC3T3, Raw264.7 and ATDC5 cells, compared to differentiated MC3T3, Raw264.7 and ATDC5 cells grown in isolated cultures.

## 4 Discussion

Our study aimed to develop a 3-dimensional cartilage-bone interface model which included osteoblasts, osteocytes, osteoclasts, and cartilage that can be used to better understand normal and disease related mechanisms of joints. During our study, Raw264.7 and MC3T3 and ATDC5 cells were successfully transfected so that their interactions during bone differentiation could be studied. The transfected cell lines were able to differentiate in a comparable manner to the parental lines. These cells were grown for up to 10 passages with no loss of fluorescence.

Furthermore, ATDC5 cells were grown on ultra-low attachment plates, which allowed them to develop into a novel cartilage spheroid. When differentiated, the spheroid expressed cartilage gene markers *Acan*, *Col2a1*, *Crtap*, *Plod2* and *Sox9* and produced Osteoprotegerin, DKK1 and Sclerostin and stained positive for GAGs and calcium. These markers have been previously demonstrated as cartilage markers in ATDC5 2D cell differentiation (17–19), and in both human 2D chondrocyte differentiation and 3D pelleted chondrocyte differentiation models (20, 21), and in porcine chondrocyte 3D alginate models (22). This data demonstrates that the ATDC5 cells are differentiating into cartilage in the spheroid. In 2D culture, the cells required 28 days for full differentiation into cartilage, while differentiation was observed by Day 21 in the spheroid. This is a novel and cost-effective approach to assessing cartilage growth and differentiation, but does use a mouse cell line, and not primary cells

Previous work by Fuller was used as the basis for the 3D bone model, and the addition of the ATDC5 cartilage sphere allowed for the design of an *in vitro* 3D BCI model (23). Imaging revealed that the ATDC5 spheroid was able to attach to the 3D bone, though the size of the spheroid was quite small in comparison to the bone. Multiple spheroids could be added to the 3D bone model to compensate for the size difference. The 3D spheroid as well as the 3D BCI stained positive for TRAP, ALP, calcium deposition and proteoglycan accumulation. Positive ALP and calcium deposition in 2D differentiated MC3T3 cells has been previously reported (24). Raw264.7 cells grown in a microfluid chip with primary osteoblasts differentiated into osteoclasts and had increased TRAP staining (14). TRAP, ALP, and calcium deposition were present in a Human 3D bone model made of differentiated primary osteoblasts and osteoclasts, developed by Visconti, et al. (25). Based on previously reported data, the positive staining that was seen in the 3D BCI model confirms both bone and cartilage formation.

The 3D BCI model produced Osteoprotegerin, which is essential for bone homeostasis (26). Sclerostin is produced by osteocytes to stop the production of bone (3, 27). The increase in sclerostin in the 3D BCI model confirms that the MC3T3 cells have fully differentiated into osteoblasts and then osteocytes. The trending increases in osteocyte markers DKK1 and FGF23 also confirm that osteocytes were embedded in the 3D BCI model. Gene expression confirmed that the 3D BCI model contained osteoblast markers *Alpl*, *Bglap*, *Col1a2*, *Mmp2*, *Runx2*, *Sparc* and *Spp1*, osteoclast markers *Acp5*, *Ctsk*, *Dcstamp*, *Ocstamp* and *Rank*, and cartilage markers *Acan*, *Crtap*, *Plod2* and *Sox9*. Upregulation of osteoblast, osteoclast and cartilage gene expression markers have all been previously reported via 2D and 3D differentiation assays (24–27). The gene expression profile in the 3D BCI model reveals that MC3T3 cells differentiated into osteoblasts and osteocytes, Raw264.7 cell differentiated into TRAP producing osteoclasts, and ATDC5 cells differentiated into cartilage. Dysregulated bone and cartilage markers are associated with disease. *Runx2* mRNA levels are increased in Ankylosing Spondylitis (28). A recently developed mouse model detected reduced *Acan* and *Col2a1* in mice with osteoarthritis (29). There is a gradual decrease in the mRNA levels of *Col2a1*, *Acan* and *Sox9* and an increase in *Runx2* associated with osteoarthritis (30). *Acp5* and *Ctsk* have been identified as markers for Rheumatoid Arthritis and are upregulated in the synovial tissue (31, 32). The ability to detect these gene expression markers in the 3D BCI model makes it suitable to interrogate inflammatory bone diseases.

We have successfully developed a mouse 3D BCI model that can be used as a screening tool to study joint disease to work in conjunction with *in vivo* screening assays. Imaging the 3D BCI model shows evidence that the Raw264.7 cells directly interacted with the differentiated MC3T3 cells and that the ATDC5 spheroid adhered to the 3D bone. The model can interrogate bone and cartilage interactions but is lacking some of the cells that reside in the joint such as synoviocytes and peripheral blood mononuclear cells (PBMCs). Experiments can be performed to add mouse PBMCs or mouse fibroblasts to better represent the joint. Because this model was developed with mouse cells, it may not reflect all aspects of human disease, but characteristics can be compared to *in vivo* mouse models. The use of cell lines instead of primary cells also creates limitations, since immortalized cells have transformed and lost some natural, biological traits (23). The mouse cell lines could be replaced with human cells, either using cell lines or primary cell cultures. But more importantly, based on our results, the joint model contains functional osteoblasts, osteoclasts, osteocytes, and chondrocytes that all interact in one system. With the 3D BCI model as a foundational model, more work can be done to use this model to establish disease models to investigate osteoarthritis and RA.

## Data Availability

All relevant data is contained within the article: The original contributions presented in the study are included in the article/supplementary material, further inquiries can be directed to the corresponding author/s.
